# Preparing for Shotgun Sequencing in Forensic Genetics—A Workflow for Analysis of Monozygotic Twins

**DOI:** 10.3390/genes17040409

**Published:** 2026-03-31

**Authors:** Olivia Luxford Meyer, Claus Børsting, Jeppe Dyrberg Andersen, Marie-Louise Kampmann

**Affiliations:** Section of Forensic Genetics, Department of Forensic Medicine, Faculty of Health and Medical Sciences, University of Copenhagen, 2100 Copenhagen, Denmark; olivia.meyer@sund.ku.dk (O.L.M.); claus.boersting@sund.ku.dk (C.B.); jeppe.dyrberg.andersen@sund.ku.dk (J.D.A.)

**Keywords:** monozygotic twins, shotgun sequencing, forensic genetics, human identification

## Abstract

Background/Objectives: Identifying the true perpetrator among monozygotic (MZ) twins has long posed a challenge in forensic genetics and for police investigations because MZ twins are likely identical in their short tandem repeat (STR) profile. In this study, we propose a workflow to address this issue, anchored in a case study involving MZ twins. Methods: The workflow includes shotgun sequencing of reference samples from one twin and trace samples, followed by a targeted amplicon-based approach (AmpliSeq™ Custom Panel) to validate the identified variants. Results: Biological traces from the crime scene, identified as blood, were available for analysis along with reference material from one twin, collected as a buccal swab. The samples underwent shotgun sequencing, and the resulting reads were aligned to the human reference genome. The sequencing yielded 2.91 and 2.85 billion mapped positions (corresponding to a breadth of coverage of 91% and 89%), with mean depths of coverage of 39.7× and 25.8× for the trace and reference samples, respectively. To minimise the risk of detecting somatic variants in the two different tissues, a stringent heterozygosity balance threshold (Hb: 0.4–0.6) was applied, and 2,047,077 single nucleotide variants (SNVs) were identified, of which 28 showed inconsistencies between the trace and reference samples. These candidate variants were subjected to validation using an AmpliSeq™ Custom Panel. Conclusions: No detectable SNV differences were observed between the reference and trace samples, and it was not possible to determine whether the trace originated from the reference twin or from his monozygotic co-twin.

## 1. Introduction

Approximately one in 250 births worldwide (0.4%) results in the birth of a monozygotic (MZ) twin pair [1]. Criminal and relationship cases involving MZ twins present a peculiar forensic genetic challenge, since the standardised short tandem repeat (STR) analysis is (usually) unable to differentiate MZ twins [2]. MZ twins originate from one single fertilised egg and share the same genetic material in the zygote. Nevertheless, postzygotic mutations may arise after the zygote splits into two embryos, and these mutations may lead to genetic differences between the twins that can be the key for individual identification [1,3,4]. Whole-genome sequencing via shotgun sequencing provides an opportunity to detect subtle genetic variations in individuals presumed to be genetically identical [1].

Previous case reports have investigated MZ twins. Weber-Lehmann et al. [5] identified five informative single nucleotide variants (SNVs) in a pair of MZ twins using shotgun sequencing, with subsequent validation via Sanger sequencing and manual inspection using the Integrative Genomics Viewer (IGV). Similarly, Yuan et al. [6] reported the detection of one SNV in the mitochondrial genome that differed between an MZ twin pair. In this case, shotgun sequencing was employed for variant screening, while the amplification refractory mutation system polymerase chain reaction (ARMS-PCR) was used for confirmation. More recently, in 2024, Sun et al. [7] identified nine SNVs that differed between a pair of MZ twins. To assess whether additional mutations accumulated over time, the researchers analysed samples from the twins at ages 27 and 33. The same nine SNVs were consistently detected at both time points, suggesting that the number of detectable post-twinning mutations remained stable over time. In 2021, Rolf and Krawczak [8] compiled six cases, including mock, criminal, and kinship investigations, in which shotgun sequencing was utilised to differentiate between MZ twins. Across these cases, between zero and 11 SNVs were identified. In 2025, van der Gaag [9] and colleagues presented a laboratory method combined with a statistical framework for discriminating MZ twins by analysing both germline and somatic variants. Their findings supported the criminal conviction of one of the twins for a criminal act. In a larger study of Icelandic twins, Jonsson et al. [10] sequenced 381 MZ twin pairs and reported a median of 14 informative SNVs and indels (tissue-specific -somatic and early post-zygotic mutations -near germline variations). A total of 39 twin pairs (10%) had more than 100 informative SNVs and indels, whereas 38 twin pairs (10%) did not differ at all. The study also investigated tissue-specific accumulation of informative variation (somatic informative variation) and found these to be more common in blood samples compared to buccal swab samples. These were only seen in a small fraction of the investigated cells, and hence the read frequency of the variant allele was often lower than expected for heterozygous genotypes (5–20% compared to the second allele).

This case study aimed to genetically differentiate between a pair of MZ twins relevant to an ongoing police investigation. Law enforcement authorities provided biological evidence from the crime scene, consisting of blood samples attributed to the perpetrator (trace sample), along with a buccal swab obtained from one of the MZ twins (reference sample). Samples from the second twin were not available. To identify genetic variations between the reference and trace samples, shotgun sequencing was employed, and the detected SNVs were subsequently analysed using Ion AmpliSeq™ Targeted Sequencing Technology (Thermo Fisher Scientific, Waltham, MA, USA). The method described here provides a workflow that may be used for similar cases involving MZ twins.

## 2. Materials and Methods

### 2.1. Samples and DNA Extraction

The samples comprised two cotton swabs collected from the crime scene, presumed to contain blood (hereafter referred to as the trace samples), and two OmniSwabs (Qiagen, Hilden, Germany) from a suspect (hereafter referred to as the reference samples). The trace samples were collected on two separate cotton swabs, which were treated as replicates and subjected to DNA extraction using the QIAamp DNA Investigator Kit (Qiagen, Hilden, Germany), following the manufacturer’s protocol for the isolation of total DNA from surface and buccal swabs. Additionally, the two OmniSwabs, also treated as replicates, were extracted in a separate laboratory using the same extraction methods in accordance with the manufacturer’s instructions (QIAamp DNA Investigator Kit, Qiagen).

### 2.2. Ethics

The study follows the policies from the Danish National Center for Ethics (https://nationaltcenterforetik.dk/ (accessed on 11 February 2025)) and the Danish Research Ethics Committees (https://videnskabsetik.dk/ (accessed on 1 February 2025)) and complies with the rules of the General Data Protection Regulation (Regulation (EU) 2016/679). According to the Danish Act on Ethics Review of Health Research Projects and Health Data Research Projects (“the Committee Act”) e.c. Section 1, method studies are exempt from ethical review. According to Danish ethical guidelines and legislation, only health-related research projects are subject to ethical approval by a research ethics committee.

Informed consent for participation is not required since this work was part of a police investigation and the biological material was made available in pseudonymised form. The police district granted the transfer and use of the data for research purposes.

### 2.3. Quantification and STR Amplification

Human DNA from all the samples was quantified using the Quantifiler™ Trio DNA Quantification Kit (Thermo Fisher Scientific, Waltham, MA, USA). STR amplifications were performed in duplicate with the GlobalFiler™ IQC PCR Amplification Kit (Thermo Fisher Scientific, Waltham, MA, USA), in accordance with the manufacturer’s instructions, using an input of 500 pg DNA. Capillary electrophoresis was conducted using the 3500xL Genetic Analyzer (Thermo Fisher Scientific, Waltham, MA, USA). To prevent contamination, the trace and reference samples were processed in separate laboratories until the completion of the amplification step.

### 2.4. Library Building and Shotgun Sequencing

Library building of both the trace and reference samples was performed using the TruSeq DNA Nano Kit (Illumina, San Diego, CA, USA) following the manufacturer’s instructions, with an input of 100 ng DNA. To minimise the risk of contamination, library preparation was conducted in separate laboratory facilities for the trace and reference samples. The number of amplification cycles was determined according to the protocol described in [11]. Following amplification, libraries were quantified for sequencing using the KAPA Library Quantification Kit (Roche, Basel, Switzerland). Finally, sequencing was carried out on two separate SP flow cells using the NovaSeq 6000 SP Reagent Kit v1.5 (150 PE, 300 cycles, Illumina, San Diego, CA, USA), with the two trace replicates and the two reference replicates sequenced separately.

### 2.5. Data Analysis of Shotgun Sequencing

Raw BCL data from the sequencing was converted into fastq files using bcl2fastq2 (version 2.20.0.422, Illumina), followed by adapter-trimming using AdapterRemoval (version 2.1.3.) [12] with a minimum trimming quality of 30, maximum N’s allowed of 30, and minimum fragment length of 30. The reads were aligned to the human reference genome GRCh38 using BWA-MEM (version 0.7.10-r789) [13]. MarkDuplicates (Picard version 2.25., Broad Institute), base recalibration and variant calling were performed using the Genome Analysis Toolkit (GATK, version 4.0.) [14], where the genotype quality (GQ) was retrieved. GQ represents an assessment of the Phred-scaled likelihood of the genotype call [13]. For quality assessment, the software FastQC (version 0.11.2) was used. To enable comparisons of variants identified in the trace and reference samples, respectively, we performed genotype calling with GATK for all the variants identified in the variant calling step. For each individual replicate, we accepted genotypes with read depth (DP) ≥ 8 and genotype quality (GQ) ≥ 10 [15]. Further, only biallelic SNVs were considered. For heterozygote genotype calls, we applied the heterozygote balance (Hb) filters of Hb = 0.4–0.6. Only genotypes that were concordant within replicates were accepted. These genotypes were then compared between the reference and trace samples.

### 2.6. Ion AmpliSeq™ Custom Panel Design

An Ion AmpliSeq™ Custom Panel was designed to reject or confirm the genotypes of SNVs found to differ between the reference and trace samples. The panel was designed with the Ion AmpliSeq™ Designer using the DNA pipeline version 7.86. We used the DNA Gene design (multi pool) for Standard DNA (375 bp). In addition to the 28 target SNVs identified through shotgun sequencing, we included 54 SNPs from the Precision ID Identity Panel (Thermo Fisher Scientific, Waltham, MA, USA) that passed all quality filters in the shotgun sequencing. The final panel design included 17 target SNVs and 54 Precision ID Identity SNPs. Eleven of the 28 target SNVs were not included because primers for these positions were deemed “undesignable”.

### 2.7. AmpliSeq™ Library Building and Sequencing

The trace and reference samples were amplified in duplicates using the Ion AmpliSeq™ Custom Panel and converted to sequencing libraries using the Ion AmpliSeq™ Library Kit 2.0 following the manufacturer’s instructions, with the exception that only half of the volumes of the reagents were used [16,17]. The libraries were sequenced in duplicates using an Ion GeneStudio™ S5 System (Thermo Fisher Scientific, Waltham, MA, USA) with the Ion S5™ Precision ID Chef & Sequencing Kit (Thermo Fisher Scientific, Waltham, MA, USA) and two separate Ion 530™ Chips (Thermo Fisher Scientific, Waltham, MA, USA). One negative control sample was included on each chip.

### 2.8. Data Analysis of Targeted Sequencing

Fastq files were exported from the S5 Torrent Server VM (Torrent Suite 5.10.1) with the FileExporter plugin (v5. 10.0.0 (963)). Alignment was performed as described for data analysis of shotgun sequencing. Genotypes for the 71 positions covered in the Ion AmpliSeq™ Custom Panel were called using GATK (version 4.0) (14). Genotypes were accepted when DP ≥ 100 and GQ ≥ 10. We accepted heterozygotes if the Hb was 0.7–0.3, and homozygotes if the noise <0.05. Similarly to shotgun sequencing, only biallelic SNVs were considered.

## 3. Results

As suspected with MZ twins, the standard forensic STR profiles from the reference samples and the trace samples were identical. Shotgun sequencing was subsequently applied to investigate potential SNV differences between the reference sample and the trace sample. Two trace samples and two reference samples were sequenced in duplicates on two flow cells using the NovaSeq 6000. The total number of reads was, on average, 711 million reads and 995 million reads for the trace and the reference samples, respectively. After alignment and filtration, the data yielded 2.91 and 2.85 billion positions (filtered for read depth ≥8 reads), corresponding to a breadth of coverage of 91% and 89% with an average read depth of 39.7 and 25.8 for the trace and reference, respectively. We detected 4.60 million and 4.42 million SNVs in the trace and reference detected in both duplicates, respectively, where the genotype differed from GRCh38.

Hb threshold stringency was systematically evaluated by applying four filtering schemes: no Hb threshold, a broad interval (0.2 ≤ Hb ≤ 0.8), a medium threshold (0.4 ≤ Hb ≤ 0.6), and a narrow interval (0.45 ≤ Hb ≤ 0.55). Reduced stringency resulted in higher numbers of filtered genotypes. E.g., for those with the broad interval (0.2 ≤ Hb ≤ 0.8), 4,060,850 genotypes were retained, whereas 1,520,385 genotypes were retained with the most stringent threshold (0.45 ≤ Hb ≤ 0.55). Correspondingly, the number of genotypes available for comparison and the number of detected inconsistencies between the trace and reference samples depended on the Hb threshold. The number of inconsistencies was 7982, 777, 28, and 6 for the four different thresholds, respectively.

Genotype comparisons across the 2,047,077 loci revealed 28 single- nucleotide loci with differing genotypes between the trace and reference samples. In all positions, the trace sample had a homozygous genotype, and the reference sample had a heterozygous genotype or vice versa. The average read depth for the 28 positions was 15.4 and 25.9 reads for the trace and reference, respectively.

**Figure 1:**
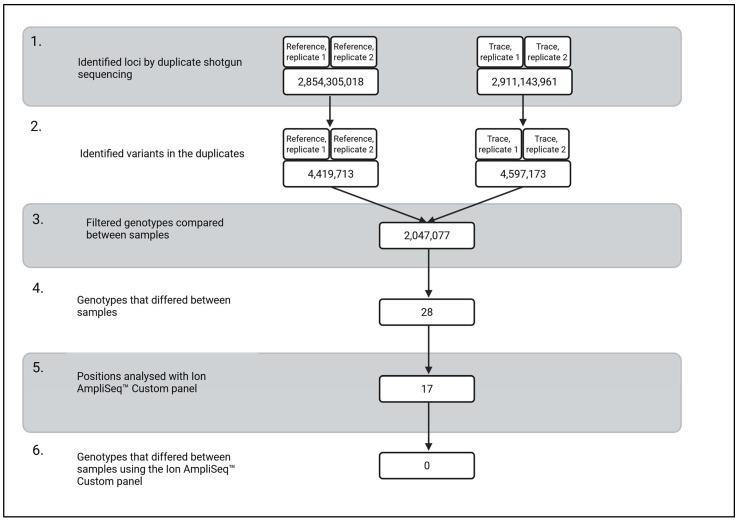
Overview of the loci, variants and genotypes in both the trace and reference sample. Step 1: The number of identified positions covered with ≥ 8 reads in both replicates of the reference and trace sample. Step 2: The number of variants identified in both replicates. Criteria for accepted genotype: Read depth ≥ 8, genotype quality ≥ 10, and only single nucleotide variants were considered. Step 3: The number of variants identified in both the reference and trace sample where 0.4 ≤ Hb ≥ 0.6. Step 4: The number of variants where the genotypes differed between the reference and trace sample. Step 5: The number of variants that are analysed by the Ion AmpliSeq™ Custom Panel. It was not possible to include 11 of the 28 identified loci in the Ion AmpliSeq™ Custom Panel. A total of 54 SNPs commonly used for human identification were also included in the Ion AmpliSeq™ Custom Panel. Step 6: The number of variants where the genotypes varied between the reference and the trace sample using the Ion AmpliSeq™ Custom Panel. Read depth ≥ 100, genotype quality ≥ 10 and 0.3 ≤ Hb ≥ 0.7.

An Ion AmpliSeq™ Custom panel was designed to confirm or reject the results from the shotgun sequencing. Of the 28 positions, 11 were not included in the panel because primers for these positions could not be designed with the AmpliSeq™ designer. The remaining 17 positions were amplified and sequenced with the custom panel (accepted if read depth ≥ 100, genotype quality ≥ 10, heterozygote balance 0.3–0.7, noise < 0.05) together with 54 commonly used human identification SNPs. The genotypes generated from the trace and reference samples with the AmpliSeq™ Custom panel were identical in all 71 positions, including the 17 positions where the genotypes differed in the shotgun sequencing data. Figure 1 summarises the key results.

## 4. Discussion

The central question of the case investigation was whether the biological trace originated from the twin who provided the reference sample or from his MZ co-twin. Our analyses were ultimately inconclusive, as no SNV differences between the trace and the reference were detected and verified using two independent methods. In this case, only the reference samples from one MZ twin were available, which made it impossible to compare shotgun sequencing data from both twins. The trace sample was of high quality, but no SNVs were detected between the reference sample and the trace sample, suggesting that either the twin providing the reference sample was in fact the source of the trace sample, or the two twins were genetically identical for the applied methods. According to the police investigation, the twin who donated the reference sample had an alibi and could therefore not have been the donor of the trace. A previous study [10] indicated that approximately 10% of MZ twin pairs are identical. Consequently, there is a risk that no informative variants will be detected in a case involving MZ twins and that shotgun sequencing will not provide additional evidence to the conventional STR profiling. A key limitation of this study was the unavailability of a reference sample from the second twin. Inclusion of such a sample would have enabled a direct comparison between two high-quality reference profiles derived from the same tissue type, thereby establishing whether any baseline genetic differences existed prior to analysis of the trace material. Nevertheless, owing to the high quality of the trace sample and the substantial amount of biological material available, it was possible to perform a robust comparison between the trace sample and the high-quality reference sample.

Because the samples derived from different tissues (blood and buccal swab, respectively), stringent heterozygote filters were applied (Hb = 0.4–0.6, read depth ≥ 8 and genotype quality ≥ 10) to exclude potential tissue-specific (somatic) variants that would likely have a more skewed heterozygote balance [10]. Any SNVs detected under these conditions would therefore be expected to have arisen post-zygotically, i.e., after the twinning event but during foetal development. However, the use of too stringent criteria may also reduce the sensitivity of detecting subtle genetic differences between MZ twins. A sample of the same body fluid as the trace (in this case, blood) from both twins would increase the power of discrimination, as it would allow the inclusion of the somatic variants in the analysis. The stringent quality parameters applied in this study, including high sequencing depth and narrow Hb thresholds, will limit the detection of true variants. However, these criteria were necessary to minimise the inclusion of somatic mutations and thereby enhance the reliability of the resulting genotype calls. A particular challenge in this study was the use of non-matching tissue types, as only a buccal swab was available as a reference sample for one twin, whereas the trace material recovered from the crime scene consisted of blood. To mitigate potential biases arising from this discrepancy and avoid false positives due to tissue-specific variation, highly stringent filtering parameters were implemented to ensure that only robust and reproducible variants were retained.

Evaluation of alternative Hb thresholds demonstrated the expected trade-off between sensitivity and specificity: with narrower thresholds yielding fewer discordant genotypes and broader thresholds increasing the number of observed differences. To reduce the risk of incorporating somatic variation [10], excessively permissive criteria were excluded. Conversely, the most restrictive thresholds were also not adopted, as they substantially reduced the number of informative variants available for comparison. Collectively, the selected filtering strategy represents a balance between minimising technical and somatic variation while retaining sufficient variants for downstream analyses.

Other sources of evidence could also be informative in discriminating MZ twins. Fingerprints and DNA methylation profiles [18,19] were shown to discriminate MZ twins. However, no fingerprints were collected in this case, and DNA methylation is known to be tissue-specific [20]; thus, it would not be suitable for discriminating MZ twins based on patterns from different tissues.

The findings of this study were inconclusive, as no discriminating SNVs between the trace sample and the reference sample from one twin were found using two independent methods. Consequently, the analysis could not determine whether the reference and the trace sample originated from the same individual or from two distinct MZ twins. However, this study provides a robust and reproducible workflow for future forensic investigations involving MZ twins, encompassing laboratory procedures, suggested parameters for data analysis, and an independent strategy for validation of identified variants. The proposed workflow is also usable in scenarios where the reference samples from both twins are unavailable, or in situations where the biological material from the crime scene is either unavailable as reference material or of unknown origin.

## Data Availability

Due to privacy restrictions, the data is not available online.

## Abbreviations

The following abbreviations are used in this manuscript:

MZ	Monozygotic twins
STR	Short tandem repeats
Hb	Heterozygosity balance
SNVs	Single nucleotide variants
GQ	Genotype quality
DP	Read depth

## References

[1] Hall J.G. (2003). Twinning. Lancet.

[2] Aourangzaib M., Chandra M., Maham R., Naz A., Malathi H., Qadeer S., Mateen R.M., Parveen R. (2024). Solving the twin paradox-forensic strategies to identify the identical twins. Forensic Sci. Int..

[3] Herranz G. (2014). The timing of monozygotic twinning: A criticism of the common model. Zygote.

[4] McNamara H.C., Kane S.C., Craig J.M., Short R.V., Umstad M.P. (2016). A review of the mechanisms and evidence for typical and atypical twinning. Am. J. Obstet. Gynecol..

[5] Weber-Lehmann J., Schilling E., Gradl G., Richter D.C., Wiehler J., Rolf B. (2014). Finding the needle in the haystack: Differentiating “identical” twins in paternity testing and forensics by ultra-deep next generation sequencing. Forensic Sci. Int. Genet..

[6] Yuan L., Chen X., Liu Z., Liu Q., Song A., Bao G., Wei G., Zhang S., Lu J., Wu Y. (2020). Identification of the perpetrator among identical twins using next-generation sequencing technology: A case report. Forensic Sci. Int. Genet..

[7] Sun W., Wang Z., Wen S., Huang A., Li H., Jiang L., Feng Q., Fan D., Tian Q., Han D. (2024). Technical strategy for monozygotic twin discrimination by single-nucleotide variants. Int. J. Leg. Med..

[8] Rolf B., Krawczak M. (2021). The germlines of male monozygotic (MZ) twins: Very similar, but not identical. Forensic Sci. Int. Genet..

[9] van der Gaag K.J., van Marion V., van den Berg R.R., Weiler N.E., Hoogenboom J., Kal A., Kayser M., de Knijff P., Laros J.F., Sijen T. (2025). Identifying a monozygotic twin brother as a donor of DNA in minimal, mixed forensic stains—A case example. Forensic Sci. Int. Genet..

[10] Jonsson H., Magnusdottir E., Eggertsson H.P., Stefansson O.A., Arnadottir G.A., Eiriksson O., Zink F., Helgason E.A., Jonsdottir I., Gylfason A. (2021). Differences between germline genomes of monozygotic twins. Nat. Genet..

[11] Gansauge M.T., Meyer M. (2013). Single-stranded DNA library preparation for the sequencing of ancient or damaged DNA. Nat. Protoc..

[12] Schubert M., Lindgreen S., Orlando L. (2016). AdapterRemoval v2: Rapid adapter trimming, identification, and read merging. BMC Res. Notes.

[13] Li H., Durbin R. (2010). Fast and accurate long-read alignment with Burrows-Wheeler transform. Bioinformatics.

[14] McKenna A., Hanna M., Banks E., Sivachenko A., Cibulskis K., Kernytsky A., Garimella K., Altshuler D., Gabriel S., Daly M. (2010). The genome analysis toolkit: A MapReduce framework for analyzing next-generation DNA sequencing data. Genome Res..

[15] Kampmann M.L., Børsting C., Jepsen A.H., Andersen M.M., Aagreen C.I., Poggiali B., Jønck C.G., Morling N., Andersen J.D. (2025). Preparing for shotgun sequencing in forensic genetics—Evaluation of DNA extraction and library building methods. Forensic Sci. Int. Genet..

[16] Buchard A., Kampmann M.L., Poulsen L., Børsting C., Morling N. (2016). ISO 17025 validation of a next-generation sequencing assay for relationship testing. Electrophoresis.

[17] van der Heijden S., de Oliveira S.J., Kampmann M.L., Børsting C., Morling N. (2017). Comparison of manual and automated AmpliSeq^TM^ workflows in the typing of a Somali population with the Precision ID Identity Panel. Forensic Sci. Int. Genet..

[18] Vidaki A., Kalamara V., Carnero-Montoro E., Spector T.D., Bell J.T., Kayser M. (2018). Investigating the epigenetic discrimination of identical twins using buccal swabs, saliva, and cigarette butts in the forensic setting. Genes.

[19] Cho M.J., Park S.J., Lee H.Y., Park J.L., Lee S.M., Kim J.Y., Lee S.Y., Kim S.Y., Jun J.K., Lee S.D. (2025). Genome-wide DNA methylome profiling to differentiate monozygotic twins. Forensic Sci. Int. Genet..

[20] Lokk K., Modhukur V., Rajashekar B., Märtens K., Mägi R., Kolde R., Koltšina M., Nilsson T.K., Vilo J., Salumets A. (2014). DNA methylome profiling of human tissues identifies global and tissue-specific methylation patterns. Genome Biol..

